# Repeat cytoreductive surgery with HIPEC for colorectal peritoneal metastases: a systematic review

**DOI:** 10.1186/s12957-024-03386-6

**Published:** 2024-04-17

**Authors:** Mina Sarofim, Ruwanthi Wijayawardana, Nima Ahmadi, David L. Morris

**Affiliations:** 1 Liver and Peritonectomy Unit, St George Hospital, Gray St, Kogarah, NSW 2217 Australia; 2 School of Medicine, University of New South Wales, Sydney, NSW Australia; 3 School of Medicine, University of Sydney, Sydney, NSW Australia

**Keywords:** Cytoreductive surgery, Colorectal cancer, Peritoneal carcinomatosis, Peritoneal metastases

## Abstract

**Background:**

Colorectal peritoneal metastases (CRPM) are present in 10–20% of patients at the time of their initial cancer diagnosis, and affects over 20% of those who develop colorectal cancer recurrence. Cytoreductive surgery (CRS) with HIPEC is firmly established as the optimal surgical treatment, but there is very little known about the benefit of repeat or iterative CRS. The aim of this review is to provide a systematic evaluation of the perioperative complications, survival outcomes and quality of life in patients undergoing repeat CRS with HIPEC for CRPM.

**Methods:**

A systematic review of PubMed, Ovid MEDLINE, EMBASE, Scopus and Cochrane databases was performed to identify all studies that reported outcomes for repeat CRS with or without HIPEC for CRPM.

**Results:**

Four hundred and ninety-three manuscripts were screened, and 15 retrospective studies were suitable for inclusion. Sample sizes ranged from 2 to 30 participants and comprised a total of 229 patients. HIPEC was used in all studies, but exact rates were not consistently stated. Perioperative morbidity was reported in four studies, between 16.7% and 37.5%. Nine studies reported mortality rate which was consistently 0%. The median overall survival after repeat CRS ranged from 20 to 62.6 months. No studies provided quality of life metrics.

**Conclusion:**

Repeat CRS for CRPM has perioperative morbidity and mortality rates comparable to initial CRS, and offers a potential survival benefit in selected patients. There is however limited high-quality data in the literature.

**Supplementary Information:**

The online version contains supplementary material available at 10.1186/s12957-024-03386-6.

## Introduction

The incidence of colorectal cancer (CRC) continues to increase dramatically, estimated by the World Health Organisation to soon exceed 3 million new cases annually [1]. Localised transcoelomic spread of malignant cells can lead to invasion of the submesothelial layer of the peritoneum resulting in metastatic nodular deposits. These are termed colorectal peritoneal metastases (CRPM), present at initial diagnosis in 10–20% of patients and up to 20% of those who develop subsequent CRC recurrence [2, 3]. Over the last two decades, cytoreductive surgery (CRS) with heated intraperitoneal chemotherapy (HIPEC) has been well studied and its role firmly established as optimal locoregional treatment of CRPM [4–6]. Combined with systemic chemotherapy, patients undergoing CRS and HIPEC can achieve a 3-survival rate of over 50%, and a median survival of 41 months. This is a radical improvement compared to the former treatment paradigm of systemic chemotherapy alone, which offered median survival of only 3–7 months [7–9]. 

Patients with peritoneal malignancy who develop recurrent abdominal disease will eventually succumb to pain, ascites, intestinal obstruction or enteric fistulae [10, 11]. Treatment is complicated by unique challenges which affect this cohort such as frequent malnutrition, prolonged chemotherapy regimens and management of extra-abdominal disease [12]. Repeat or iterative CRS refers to a subsequent surgical procedure to remove recurrent peritoneal disease in patients who have undergone initial CRS. The rationale for this approach is that reduction of tumour burden avoids complications of disease progression, improves quality of life and extends patient survival. The indications, outcomes and benefit of repeat CRS with or without HIPEC for recurrent peritoneal disease has gradually become an area of increasing interest [13–15]. 

The existing literature examining repeat CRS has demonstrated its safety, but is based on small heterogenous cohorts of peritoneal tumours – ovarian, colorectal, appendiceal pseudomyxoma peritonei or mesothelial – which is not accurately generalisable to patients with CRPM; each tumour has inherent biological and behavioural differences [16–18]. Developing surgical treatment guidelines for recurrent CRPM, accurately balancing the potential risk-versus-benefit of repeat CRS, and optimising patient selection are therefore difficult to address due to the lack of high-quality evidence. We hypothesise that repeat CRS can offer valuable disease control and survival benefit, with acceptable morbidity and mortality rates. The aim of this review is to provide a systematic evaluation of survival outcomes, complications and quality of life indicators in patients undergoing repeat CRS with HIPEC for CRPM.

## Methods

### Literature search

We conducted a systematic review of the literature to identify studies which investigated the outcomes of repeat CRS in CRPM, in accordance with the Preferred Reporting Items for Systematic Reviews and Meta-Analyses (PRISMA) guidelines [19]. A comprehensive search was conducted in the following electronic databases: PubMed, Ovid MEDLINE, EMBASE, Scopus and Cochrane Central Register of Controlled trials. Individual search strategies were tailored to each database using the following Medical Subjects Headings (MeSH; in bold), Boolean operators (‘AND’, ‘OR’) and key terms:

1. **Cytoreduction Surgical Procedures** OR Peritonectomy OR Debulking surgery.
2. **Hyperthermic Intraperitoneal Chemotherapy** OR HIPEC OR Intraperitoneal chemotherapy.
3. **Peritoneal Neoplasms** OR Peritoneal metastases OR Peritoneal Carcinomatosis.
4. **#1 OR #2 OR #3**
5. **Reoperation** OR Secondary operation OR Iterative.
6. #4 AND #5

The search was performed without language or date restrictions to produce all publications up to November 2023. To further identify possible studies, reference lists of identified systematic reviews and relevant articles were hand searched.

### Inclusion and exclusion criteria

To be included, articles had to describe studies meeting the following criteria:

1. Adult patients (age > 18).
2. Participants underwent repeat/secondary cytoreductive surgery.
3. Report at least one of the following endpoints for CRPM: median or overall survival, disease-free survival, postoperative morbidity or mortality, or quality of life.
4. Published in a peer-reviewed journal.

Exclusion criteria were non-human studies, case reports (or subgroup of only one patient), letters, editorials, conference abstracts, non-English publications, or studies that did not report sufficient CRPM data for extraction.

### Quality assessment

The methodological quality of the studies was assessed using a modified Newcastle-Ottawa Scale (NOS) for observational studies. Studies with NOS scores of 7 were considered high quality [20]. 

### Selection of papers

Manuscript assessment was performed independently by two reviewers (MS and NA) using a standardized, pre-piloted form. This included study design, inclusion and exclusion criteria, baseline characteristics, primary or repeat cytoreductive procedures, and endpoint outcomes.

### Data extraction and statistical analysis

Data extraction included PCI and CC score, follow-up duration, and outcomes: median or overall survival, disease-free survival, postoperative morbidity and mortality, or quality of life. Morbidity was defined using Clavien-Dindo Classification [21] Grade III (requiring surgical, endoscopic or radiological intervention) or Grade IV (requiring intensive care or organ support). Outcome data was recorded in its reported format of median (range), mean (standard deviation) or absolute percentages. If required, median survival was alternatively extrapolated from Kaplan-Meier survival curves. Data was also extracted on confounding variables such as adjuvant or neoadjuvant chemotherapy, or other novel treatments if reported. All statistical analysis was performed using SPSS version 24 (IBM, USA). A *p*-value < 0.05 was considered significant.

### Ethical considerations

This study did not require ethics approval as it synthesised published data. It was registered in the PROSPERO database.

## Results

### Study characteristics

**Fig. 1:**
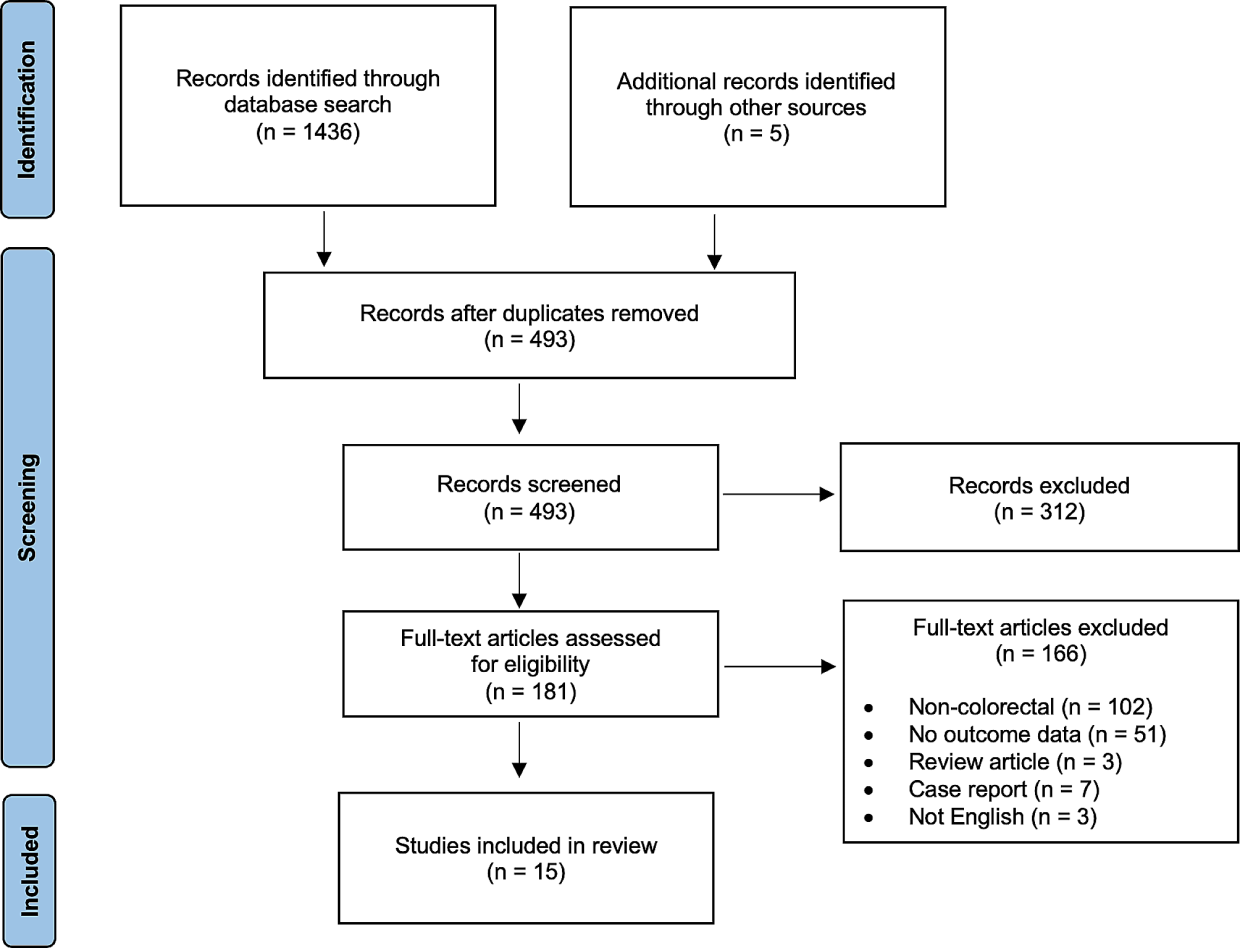
PRISMA flowchart of study selection

After removal of duplicates, a total of 493 articles were retrieved from the database search including five additional references found through review of reference lists. A flowchart of the study selection is shown in Fig. 1. Once article abstracts were screened, 312 were excluded leaving 181 eligible. After full texts were assessed, an additional 166 were excluded including a multi-institutional study to avoid reporting the same patients repeatedly [22]. Fifteen articles were therefore included in the analysis with a pooled total of 229 patients. Study sample sizes ranged from 2 to 30 participants. Only one study had a median/mean age above 60. Demographic information is listed in Table 1.

**Table 1:** Characteristics of included studies

Author	Year	Study design	CRPM specific	Sample size	Mean age (years)	Males (%)
**Portilla**et al. [15]	1999	R	Y	18	55.3	72.2
**Glehen**et al. [25]	2004	R	Y	26	.	.
**Bijelic**et al. [26]	2008	R	Y	18	.	.
**Bretcha-Boix**et al. [27]	2010	R	Y	2	55.5∼	40∼
**Cashin**et al. [28]	2012	R	Y	8	.	.
**Votanopoulos**et al. [29]	2012	R	N	4	46.4 (SD 11.1)∼	43.5∼
**Chua**et al. [30]	2013	R	N	11	53 (R 19–79) ^m^	49∼
**Williams**et al. [31]	2014	R	Y	18	52.4 ^m^	38.9
**Choudry**et al. [23]	2019	R	N	29	52.2 (SD 10.6)∼	.
**Jost**et al. [32]	2020	R	Y	9	47	33
**Laks**et al. [24]	2021	R	Y	30	58.7	26.7
**Paasch**et al. [33]	2021	R	N	7	58.1	42.9
**Sutton**et al. [18]	2021	R	N	18	53 (R 44–63) ^m^	94.4
**Valenzuela**et al. [34]	2022	R	N	16	52.6∼	43.2∼
**Pasqual**et al. [35]	2023	R	N	15	61.65 (SD 11.44)∼	.

CRPM = colorectal peritoneal metastases.

R = retrospective.

Y = yes, N = no.

∼ = of larger peritoneal malignancy cohort.

m = median instead of mean.

SD = standard deviation, R = range

### Quality of included studies

There were no prospective or randomised studies, all were retrospective observational studies of fair quality (Supplementary Table 1). Eight of the studies exclusively evaluated CRPM patients, the other seven reported a heterogenous cohort of peritoneal tumours with subgroup analysis by primary tumour type. The exact duration of follow up was not frequently reported in the studies.

### Perioperative outcomes

Perioperative data is summarised in Table 2. The median PCI score at repeat CRS procedure was reported in only five papers, ranging from 5 to 10. The use of HIPEC was described in all studies but the exact proportion of patients receiving it was only explicit in four studies: 28.6%, 72.2%, 94.4% and 100% respectively. Ability to achieve complete cytoreduction score 0 or 1 was reported in four studies, and two of these were 100%.

**Table 2:** Perioperative outcomes of included studies

Author	Median PCI	HIPEC used	CC 0–1 (%)	Morbidity (%)	Mortality (%)
**Portilla**et al. [15]	.	.	61.1	.	0
**Glehen**et al. [25]	.	.	.	.	.
**Bijelic**et al. [26]	.	.	100	.	.
**Bretcha-Boix**et al. [27]	.	100	.	.	0
**Cashin**et al. [28]	.	.	.	.	0
**Votanopoulos**et al. [29]	.	.	.	.	.
**Chua**et al. [30]	.	.	.	.	0
**Williams**et al. [31]	5 (R 1–13)	72.2	100	16.7	0
**Choudry**et al. [23]	.	.	.	.	.
**Jost**et al. [32]	8	.	.	.	0
**Laks**et al. [24]	10 (R 2–28)	.	.	32.1	.
**Paasch**et al. [33]	R 7–10	28.6	.	.	0
**Sutton**et al. [18]	7 (R 5–12)	94.4	83.3	22.2	0
**Valenzuela**et al. [34]	.	.	.	37.5	0
**Pasqual**et al. [35]	.	.	.	.	.

PCI = peritoneal carcinomatosis index.

HIPEC = heated intraperitoneal chemotherapy.

CC = completeness of cytoreduction score.

R = range

Morbidity defined as Clavien-Dindo Grade III or IV was reported in four studies ranging from 16.7 to 37.5%. Operative mortality rate was reported in nine studies, consistently 0%. The use of perioperative intraperitoneal chemotherapy or adjuvant chemotherapy was not uniformly described.

### Survival outcomes

Choudry et al. [23] and Laks et al. [24] reported median disease-free survival of 9.1 months and 8.7 months respectively, and the remaining 13 studies reported overall survival which is summarised in Table 3. The longest median survival was 62.6 months (range 25.3–99.9) [18], and the shortest median survival was 20 months [15]. 

**Table 3:** Long-term outcomes of included studies

Author	Median DFS (months)	Median overall survival (months)	QOL outcomes
**Portilla**et al. [15]		20	.
**Glehen**et al. [25]	.	57.6 ^&^	.
**Bijelic**et al. [26]	.	39	.
**Bretcha-Boix**et al. [27]	.	> 18 ^m^	.
**Cashin**et al. [28]		23 (R 9–98)	.
**Votanopoulos**et al. [29]	.	55.7 (R 0.3-110.2)	.
**Chua**et al. [30]	.	23 (R 16.9–28.3)	.
**Williams**et al. [31]		22.6	.
**Choudry**et al. [23]	9.1 (R 3.9–14.3)	.	.
**Jost**et al. [32]	.	40 ^m^ (SD 12)	.
**Laks**et al. [24]	8.7 (R 1.2–26.3)	.	.
**Paasch**et al. [33]		R 16–87	.
**Sutton**et al. [18]		62.6 (R 25.3–99.9)	.
**Valenzuela**et al. [34]	.	40.1	.
**Pasqual**et al. [35]	.	21 ^#^	.

DFS = disease free survival.

QOL = quality of life.

R = range.

m = mean, SD = standard deviation.

& = from initial CRS procedure.

# calculated using Kaplan-Meier survival analysis

### Quality of life outcomes

No studies provided data to evaluate patient quality of life following repeat CRS.

## Discussion

Initiated by the work of Sugarbaker in the 1990s, there has been a paradigm shift over the last two decades towards more radical surgical management of CRPM. For patients with good performance status, initial CRS is the gold standard treatment to remove macroscopic tumour deposits [5, 6]. The peritoneal-plasma barrier aids survival and growth of microscopic neoplastic cells, which poses an obstacle to systemic chemotherapy penetration. The addition of HIPEC during CRS has recently been challenged by a randomised phase III trial, but nonetheless is commonly used to target this residual disease. Patients with CRPM are now experiencing dramatically improved survival following initial CRS and HIPEC, over 50% remaining alive after 3 years post operatively [8, 36]. When combined with estimates that the global burden of CRC will increase by 63% over the coming two decades, it is expected that an increasing number of patients will live long enough to develop disease recurrence despite the use of adjuvant chemotherapy [1]. 

The optimal management of recurrent CRPM and role of repeat CRS has not been definitively established in any international guidelines due to the unclear benefit and limited data available. This review provides a much-needed systematic assessment and up to date evaluation of the literature. Our results are based on a pooled total of 229 patients who underwent repeat CRS and HIPEC for CRPM, and supports the proposition that a valuable survival benefit is achievable. We propose that achieving a positive benefit of repeat CRS and HIPEC is challenging, but hinges on meticulous patient selection, centralisation to specialist peritoneal malignancy units, and coordination by an experienced surgical oncology multi-disciplinary team.

Long-term survival following initial CRS and HIPEC for CRPM is negatively affected by high PCI score, incomplete cytoreduction, high tumour grade and poor performance status [27, 29]. A previous systematic review reported recurrent disease after initial CRS and HIPEC occurs in 22.5–82% of patients despite a majority receiving adjuvant treatment [37]. Importantly, disease free interval must be considered when assessing suitability for repeat CRS and HIPEC. This is not only a measure of initial surgical success but also adds critically valuable prognostic information. Pragmatically this reflects aggressiveness of tumour biology, disease progression and response to treatment, which foretells trajectory of further radical treatment. Unlike those who present with an early recurrence, a longer disease-free interval after initial CRS and HIPEC has repeatedly been shown to independently predict longer survival following repeat CRS and HIPEC [23, 29, 30, 37]. This reiterates that patients with slow tumour progression are prime candidates to benefit from repeat CRS and HIPEC.

Braam et al. [38] have shown in their 287 patient cohort that surgical treatment of CRC recurrence following initial CRS and HIPEC – which included liver resection, pulmonary resection or repeat CRS and HIPEC – provides significantly longer survival when compared to the only remaining alternative of palliative treatment, median of 42.9 months versus 11.8 months respectively. A key issue raised however is the potential hazards associated with perioperative morbidity and mortality of repeat CRS and HIPEC. Based on our results, the major complication rate acceptably ranged from 16.7 to 37.5%, and mortality rate was 0%. Two of the four relevant studies in our review claimed 100% complete cytoreduction [26, 31]. These risk-benefit figures are not only comparable to initial CRS and HIPEC, but also to a multi-institutional review of repeat CRS and HIPEC for CRPM; therefore this should not present a barrier to decision-making in patients with resectable intra- or extra-abdominal disease [22]. Furthermore, advances in surgical techniques and perioperative care will no doubt continue to make repeat CRS and HIPEC safer [23, 24]. 

The role of repeat CRS becomes clearer by evaluating alternative proactive approaches of managing CRPM – prior to the onset of signs and symptoms – which have so far have yielded mixed results. The *COLOPEC* randomised trial investigated the use of oxaliplatin HIPEC in patients with T4 or perforated CRC, but this did not alter the rate of subsequent peritoneal metastases [39]. Another randomised trial *PROPHYLOCHIP-PRODIGE 15* assigned patients at risk of CRPM to either surveillance or second-look surgery plus HIPEC (oxaliplatin or mitomycin C), but this also did not show a difference in disease-free survival [40]. Most recently, the randomised *HIPECT4* trial investigated the role of mitomycin C in T4 colon tumours which did show a significant reduction in 3-year peritoneal recurrence rates, but did not change disease free interval or overall survival [41]. Alongside surgical advances, oncological treatments may also allow more patients to benefit from repeat CRS and HIPEC. The use of neoadjuvant chemotherapy prior to initial CRS has been shown to improve 5-year survival for patients with low volume CRPM, which could be further evaluated for a similar efficacy in repeat CRS procedures [42]. Additionally, emerging use of patient-derived tumour organoids as ex vivo models can preserve the original tumour microenvironment, act as biomarkers, and generate drug efficacy data to improve choice of HIPEC or adjuvant chemotherapy agents [43]. 

A significant strength of this review is the breadth of the systematic search strategy. This was utilised to capture outcome data for all peritoneal malignancy patients, then full-text manuscripts were manually evaluated for explicit or subgroup data on CRPM. Several of the included manuscripts were identified in this manner which may have been missed if the strategy focused only on CRC. This addressed one of the weaknesses of repeat CRS literature: CRPM should be viewed as a unique pathological entity, yet most of the survival data for published combines CRC with appendiceal or other peritoneal malignancy tumours. Such data is difficult to clinically apply, as unsurprisingly appendiceal and colorectal tumours behave differently and ultimately confer differing prognoses [32, 44]. A further strength is our finding that no studies to date have reported on quality of life after repeat CRS for CRPM, which presents a focus of future research using validated tools such as the 15-item quality of recovery (QoR-15) scale [45]. This would reveal novel information on the patient-centred outcomes of repeat CRS.

This review is limited by the reliance on small retrospective cohorts. The rarity of this condition makes randomised trials, or even true prospective studies, difficult to orchestrate [46]. Additionally, heterogeneity of data for the same outcome (for example, medians versus means versus range) resulted in only a descriptive evaluation of the included studies rather than a true pooled analysis. Another limitation is the lack of data within studies regarding possible confounding variables, such as timing/duration of systemic treatment regimes, clinicopathological features, serum tumour markers and prevalence of extra-abdominal disease.

## Conclusion

Repeat CRS and HIPEC for CRPM offers a survival benefit in well selected patients, particularly in the absence of effective alternative treatment options. Perioperative morbidity and mortality rates are acceptable, and comparable to initial CRS procedures. The literature consists of small retrospective cohorts, hence further prospective studies would be valuable, including a focus on quality of life metrics which may provide a novel patient-centred perspective.

### Electronic supplementary material

Below is the link to the electronic supplementary material.

Supplementary Material 1

## Data Availability

All data analysed during this study are included in this published article.

## References

[1] Eileen M, Melina A, Gini A, Lorenzoni V, Cabasag CJ, Mathieu L (2023). Global burden of colorectal cancer in 2020 and 2040: incidence and mortality estimates from GLOBOCAN. Gut.

[2] Ceelen WP, Bracke ME (2009). Peritoneal minimal residual disease in colorectal cancer: mechanisms, prevention, and treatment. Lancet Oncol.

[3] van Gestel YRBM, de Hingh IHJT, van Herk-Sukel MPP, van Erning FN, Beerepoot LV, Wijsman JH (2014). Patterns of metachronous metastases after curative treatment of colorectal cancer. Cancer Epidemiol.

[4] Cao C, Yan TD, Black D, Morris DL (2009). A systematic review and Meta-analysis of cytoreductive surgery with Perioperative Intraperitoneal Chemotherapy for Peritoneal Carcinomatosis of Colorectal Origin. Ann Surg Oncol.

[5] Sugarbaker PH (1999). Management of peritoneal-surface malignancy: the surgeon’s role. Langenbeck’s Archives Surg.

[6] Verwaal VJ, van Ruth S, de Bree E, van Sloothen GW, van Tinteren H, Boot H (2003). Randomized trial of cytoreduction and hyperthermic intraperitoneal chemotherapy versus systemic chemotherapy and palliative surgery in patients with peritoneal carcinomatosis of colorectal cancer. J Clin Oncol.

[7] Chua TC, Esquivel J, Pelz JO, Morris DL (2013). Summary of current therapeutic options for peritoneal metastases from colorectal cancer. J Surg Oncol.

[8] Quénet F, Elias D, Roca L, Goéré D, Ghouti L, Pocard M (2021). Cytoreductive surgery plus hyperthermic intraperitoneal chemotherapy versus cytoreductive surgery alone for colorectal peritoneal metastases (PRODIGE 7): a multicentre, randomised, open-label, phase 3 trial. Lancet Oncol.

[9] Vierra MA, Morgan RB, Eng OS. (2022) Advances in therapeutics for peritoneal metastases from colorectal cancer: a narrative review. Dig Med Res. 5.

[10] Chua TC, Moran BJ, Sugarbaker PH, Levine EA, Glehen O, Gilly FN (2012). Early- and long-term outcome data of patients with pseudomyxoma peritonei from appendiceal origin treated by a strategy of cytoreductive surgery and hyperthermic intraperitoneal chemotherapy. J Clin Oncol.

[11] Kranenburg O, van der Speeten K, de Hingh I (2021). Peritoneal metastases from Colorectal Cancer: defining and addressing the challenges. Front Oncol.

[12] Aoyama T, Oba K, Honda M, Sadahiro S, Hamada C, Mayanagi S (2017). Impact of postoperative complications on the colorectal cancer survival and recurrence: analyses of pooled individual patients’ data from three large phase III randomized trials. Cancer Med.

[13] Järvinen P, Järvinen HJ, Lepistö A (2010). Survival of patients with pseudomyxoma peritonei treated by serial debulking. Colorectal Dis.

[14] Mogal H, Chouliaras K, Levine EA, Shen P, Votanopoulos KI (2016). Repeat cytoreductive surgery with hyperthermic intraperitoneal chemotherapy: review of indications and outcomes. J Gastrointest Oncol.

[15] Portilla AG, Sugarbaker PH, Chang D (1999). Second-look surgery after cytoreduction and intraperitoneal chemotherapy for peritoneal carcinomatosis from Colorectal Cancer: analysis of Prognostic features. World J Surg.

[16] Mercier F, Dagbert F, Pocard M, Goéré D, Quenet F, Wernert R (2019). Recurrence of pseudomyxoma peritonei after cytoreductive surgery and hyperthermic intraperitoneal chemotherapy. BJS Open.

[17] Morice P, Dubernard G, Rey A, Atallah D, Pautier P, Pomel C (2003). Results of interval debulking surgery compared with primary debulking surgery in advanced stage ovarian cancer. J Am Coll Surg.

[18] Sutton PA, O’Dwyer ST, Barriuso J, Aziz O, Selvasekar CR, Renehan AG (2021). Indications and outcomes for repeat cytoreductive surgery and heated intra-peritoneal chemotherapy in peritoneal surface malignancy. Surg Oncol.

[19] Page MJ, McKenzie JE, Bossuyt PM, Boutron I, Hoffmann TC, Mulrow CD (2021). The PRISMA 2020 statement: an updated guideline for reporting systematic reviews. BMJ.

[20] Wells GA, Shea B, O’Connell D, Peterson J, Welch V, Losos M et al. (2000) The Newcastle-Ottawa Scale (NOS) for assessing the quality of nonrandomised studies in meta-analyses.

[21] Dindo D, Cuesta MA, Bonjer HJ (2014). The clavien–dindo classification of Surgical complications. Treatment of postoperative complications after Digestive surgery.

[22] Alzahrani NA, Valle SJ, Fisher OM, Sugarbaker PH, Yonemura Y, Glehen O (2019). Iterative cytoreductive surgery with or without hyperthermic intraperitoneal chemotherapy for colorectal peritoneal metastases: a multi-institutional experience. J Surg Oncol.

[23] Choudry HA, Bednar F, Shuai Y, Jones HL, Pai RK, Pingpank JF (2019). Repeat cytoreductive surgery-hyperthermic intraperitoneal chemoperfusion is feasible and offers Survival Benefit in Select patients with peritoneal metastases. Ann Surg Oncol.

[24] Laks S, Schtrechman G, Adileh M, Ben-Yaacov A, Purim O, Ivanov V (2021). Repeat cytoreductive surgery and intraperitoneal chemotherapy for Colorectal Cancer Peritoneal recurrences is safe and efficacious. Ann Surg Oncol.

[25] Glehen O, Kwiatkowski F, Sugarbaker PH, Elias D, Levine EA, Simone MD (2004). Cytoreductive surgery combined with Perioperative Intraperitoneal Chemotherapy for the management of peritoneal carcinomatosis from Colorectal Cancer: a multi-institutional study. J Clin Oncol.

[26] Bijelic L, Yan TD, Sugarbaker PH (2008). Treatment failure following complete cytoreductive surgery and perioperative intraperitoneal chemotherapy for peritoneal dissemination from colorectal or appendiceal mucinous neoplasms. J Surg Oncol.

[27] Bretcha-Boix P, Farré-Alegre J, Sureda M, Dussan C, Pérez Ruixo JJ, Masllorens AB (2010). Cytoreductive surgery and perioperative intraperitoneal chemotherapy in patients with peritoneal carcinomatosis of colonic origin: outcomes after 7 years’ experience of a new centre for peritoneal surface malignancies. Clin Transl Oncol.

[28] Cashin PH, Graf W, Nygren P, Mahteme H (2012). Cytoreductive surgery and intraperitoneal chemotherapy for colorectal peritoneal carcinomatosis: prognosis and treatment of recurrences in a cohort study. Eur J Surg Oncol (EJSO).

[29] Votanopoulos KI, Ihemelandu C, Shen P, Stewart JH, Russell GB, Levine EA (2012). Outcomes of repeat cytoreductive surgery with hyperthermic intraperitoneal chemotherapy for the treatment of peritoneal surface malignancy. J Am Coll Surg.

[30] Chua TC, Quinn LE, Zhao J, Morris DL (2013). Iterative cytoreductive surgery and hyperthermic intraperitoneal chemotherapy for recurrent peritoneal metastases. J Surg Oncol.

[31] Williams B, Alzahrani N, Chan D, Chua T, Morris D (2014). Repeat cytoreductive surgery (CRS) for recurrent colorectal peritoneal metastases: yes or no?. Eur J Surg Oncol (EJSO).

[32] Jost E, Mack LA, Sideris L, Dube P, Temple W, Bouchard-Fortier A (2020). Evaluation of repeat cytoreductive surgery and heated intraperitoneal chemotherapy for patients with recurrent peritoneal carcinomatosis from appendiceal and colorectal cancers: a multicentre Canadian study. Can J Surg.

[33] Paasch C, De Santo G, Gamal-Eldin HN, Hünerbein M (2021). Repeated cytoreductive surgery and hyperthermic intraperitoneal chemotherapy in patients with peritoneal carcinomatosis: a retrospective cohort study. Annals Med Surg.

[34] Valenzuela CD, Levine EA, Mangieri CW, Gawdi R, Moaven O, Russell G (2022). Repeat cytoreductive surgery with hyperthermic intraperitoneal chemotherapy for cancers with peritoneal metastasis: a 30-year institutional experience. Ann Surg Oncol.

[35] Pasqual EM, Londero AP, Robella M, Tonello M, Sommariva A, De Simone M (2023). Repeated cytoreduction combined with hyperthermic intraperitoneal chemotherapy (HIPEC) in selected patients affected by peritoneal metastases: Italian PSM Oncoteam evidence. Cancers.

[36] Li J, Wang AR, Chen XD, Zhang YX, Pan H, Li SQ (2022). Effect of hyperthermic intraperitoneal chemotherapy in combination with cytoreductive surgery on the prognosis of patients with colorectal cancer peritoneal metastasis: a systematic review and meta-analysis. World J Surg Oncol.

[37] van Oudheusden TR, Nienhuijs SW, Luyer MD, Nieuwenhuijzen GA, Lemmens VE, Rutten HJ (2015). Incidence and treatment of recurrent disease after cytoreductive surgery and intraperitoneal chemotherapy for peritoneally metastasized colorectal cancer: a systematic review. Eur J Surg Oncol.

[38] Braam HJ, van Oudheusden TR, de Hingh IH, Nienhuijs SW, Boerma D, Wiezer MJ (2014). Patterns of recurrence following complete cytoreductive surgery and hyperthermic intraperitoneal chemotherapy in patients with peritoneal carcinomatosis of colorectal cancer. J Surg Oncol.

[39] Klaver CEL, Wisselink DD, Punt CJA, Snaebjornsson P, Crezee J, Aalbers AGJ (2019). Adjuvant hyperthermic intraperitoneal chemotherapy in patients with locally advanced colon cancer (COLOPEC): a multicentre, open-label, randomised trial. Lancet Gastroenterol Hepatol.

[40] Goéré D, Glehen O, Quenet F, Guilloit J-M, Bereder J-M, Lorimier G (2020). Second-look surgery plus hyperthermic intraperitoneal chemotherapy versus surveillance in patients at high risk of developing colorectal peritoneal metastases (PROPHYLOCHIP–PRODIGE 15): a randomised, phase 3 study. Lancet Oncol.

[41] Arjona-Sánchez A, Espinosa-Redondo E, Gutiérrez-Calvo A, Segura-Sampedro JJ, Pérez-Viejo E, Concepción-Martín V (2023). Efficacy and Safety of Intraoperative Hyperthermic Intraperitoneal Chemotherapy for locally advanced Colon cancer: a phase 3 Randomized Clinical Trial. JAMA Surg.

[42] Flood MP, Kong JCH, Wilson K, Mohan H, Waters PS, McCormick JJ (2022). The impact of Neoadjuvant Chemotherapy on the Surgical Management of Colorectal Peritoneal metastases: a systematic review and Meta-analysis. Ann Surg Oncol.

[43] Erali RA, Forsythe SD, Gironda DJ, Schaaf CR, Wajih N, Soker S (2023). Utilizing patient-derived organoids in the management of Colorectal Cancer with peritoneal metastases: a review of current literature. J Gastrointest Cancer.

[44] Tabrizian P, Shrager B, Jibara G, Yang M-J, Romanoff A, Hiotis S (2014). Cytoreductive surgery and hyperthermic intraperitoneal chemotherapy for peritoneal carcinomatosis: outcomes from a single Tertiary Institution. J Gastrointest Surg.

[45] Myles PS, Shulman MA, Reilly J, Kasza J, Romero L (2022). Measurement of quality of recovery after surgery using the 15-item quality of recovery scale: a systematic review and meta-analysis. Br J Anaesth.

[46] Sarofim M (2023). The quest for excellence in surgical research. Surgeon.

