# Supplementary material for: Repeat cytoreductive surgery with HIPEC for colorectal peritoneal metastases: a systematic review
Source: World J Surg Oncol. 2024 Apr 17;22:99. doi: 10.1186/s12957-024-03386-6 (PMC11022433; doi:10.1186/s12957-024-03386-6)
Supplement: Supplementary file 1 — Supplementary Material 1 [file 12957_2024_3386_MOESM1_ESM.docx]

**Supplementary Table 1.** **Quality assessment of the included observational studies using a Modified Newcastle-Ottowa Scale.**

| **Study** | **Selection** | | | **Comparability** | **Outcome** | | **Total** |
| --- | --- | --- | --- | --- | --- | --- | --- |
|  | **Representativeness**  **(*)** | **Selection of non-exposed cohort (*)** | **Ascertainment of exposure (*)** | **(**)** | **Assessment of outcome (*)** | **Adequacy of follow up (*)** | **(7*)** |
| Portilla et al, 1999 | * | - | * | - | * | * | 4 |
| Glehen et al, 2004 | * | - | * | - | * | * | 4 |
| Bijelic et al, 2008 | * | - | * | - | * | * | 4 |
| Bretcha-Boix et al, 2010 | * | - | * | - | * | * | 4 |
| Cashin et al, 2012 | * | - | * | - | * | * | 4 |
| Votanopoulos et al, 2012 | * | - | * | - | * | * | 4 |
| Chua et al, 2013 | * | - | * | - | * | * | 4 |
| Williams et al, 2014 | * | - | * | - | * | * | 4 |
| Choudry et al, 2019 | * | - | * | ** | * | - | 5 |
| Jost et al, 2020 | * | - | * | - | * | * | 4 |
| Laks et al, 2021 | * | - | * | ** | * | - | 5 |
| Paasch et al, 2021 | * | - | * | - | * | * | 4 |
| Sutton et al, 2021 | * | - | * | - | * | * | 4 |
| Valenzuela et al, 2022 | * | - | * | - | * | * | 4 |
| Pasqual et al, 2023 | * | - | * | - | * | * | 4 |
